# Traumatic Brain Injury and Delayed Sequelae: A Review - Traumatic Brain Injury and Mild Traumatic Brain Injury (Concussion) are Precursors to Later-Onset Brain Disorders, Including Early-Onset Dementia

**DOI:** 10.1100/tsw.2007.269

**Published:** 2007-11-12

**Authors:** Michael A. Kiraly, Stephen J. Kiraly

**Affiliations:** ^1^ Department of Physiology, Faculty of Medicine, University of Toronto, Canada; ^2^ Department of Psychiatry, University of British Columbia, Canada

**Keywords:** Alzheimer’s, Parkinson’s, traumatic brain injury, concussion, amyloid, cognition

## Abstract

Brain injuries are too common. Most people are unaware of the incidence of and horrendous consequences of traumatic brain injury (TBI) and mild traumatic brain injury (MTBI). Research and the advent of sophisticated imaging have led to progression in the understanding of brain pathophysiology following TBI. Seminal evidence from animal and human experiments demonstrate links between TBI and the subsequent onset of premature, psychiatric syndromes and neurodegenerative diseases, including Alzheimer's disease (AD) and Parkinson's disease (PD). Objectives of this summary are, therefore, to instill appreciation regarding the importance of brain injury prevention, diagnosis, and treatment, and to increase awareness regarding the long-term delayed consequences following TBI.

> *With respect to the phenomenology of MTBI, there are believers and non-believers. You don't convert until it happens to you or someone close to you - William D. Singer, Harvard Professor of Neurology and Pediatrics, 2002*

Trauma to the head is dangerous – this needs no belaboring. *Dementia pugilistica* is a well-documented, organic brain syndrome, complete with heavy amyloid deposits[1]. Many people are not aware that even less severe head trauma, such as those sustained in sports, minor accidents, and even combat, have deleterious effects and are responsible for reducing brain reserve and resistance to a variety of brain disorders with delayed onset. Traumatic brain injury (TBI), especially its less severe and misunderstood variant, mild traumatic brain injury (MTBI) or concussion, have been aptly called the *silent epidemic*[2]. MTBI may be serious, but the term "mild" serves to erroneously lessen concern.

Studies have recently emerged that offer convincing evidence for a strong positive correlation between TBI and common neuropsychiatric disorders. For example, studies indicate that TBI and MTBI are epigenetic risk factors for organic personality changes[3,4,5,6], schizophrenia[16,17], depression[7,8,9,10,11], mania[12,13], Parkinson's disease (PD)[20,21], and anxiety disorders[14], which include obsessive-compulsive disorder[15], and post-traumatic stress disorder (PTSD)[18,19]. MTBI is more likely to be associated with PTSD than more severe TBI involving loss of consciousness (LOC). For a comprehensive review, refer to Van Reekum et al.[22]. Recently, data suggest that TBI has been identified as a strong positive predictor of developing other brain disorders, such as dementia, including Alzheimer's disease (AD)[23,24,30].

Head injuries are common but, for complex reasons, medical education about diagnosis and treatment does not mirror the high prevalence of this disorder. New research is of great interest to the general physician, as well as to the geriatric specialist, because TBI remains the leading cause of serious morbidity and mortality among young adults and children[23,24,30].

## THE EXTENT OF THE PROBLEM: EPIDEMIOLOGY

TBI is one of the most common neurological disorders with an incidence of 180/100,000. Disorders arising from TBI are more numerous than any other neurologic disorder with the exception of headache[26]. Such statistics are even more poignant when one considers that approximately 15% of those afflicted with TBI will be persistently symptomatic[25]. Incredibly, the cumulative impact of such disorders is equal to the annual incidence of PD, multiple sclerosis, Guillain-Barre syndrome, motor neuron disease, and myasthenia gravis *combined*. Motor vehicle accidents, falls, assaults, and various sports-related activities result in TBI that affects about 7 million individuals each year in North America[27]. Before 1999, in the U.S., 2.5–6.5 million people lived with the long-term consequences of TBI[28], however, more recent reports indicate that this has increased to an estimated 5.3 million. In other words, a little more than 2% of the entire U.S. population currently lives with disabilities resulting from TBI[29], which translates to a TBI-associated cost in the U.S. of an estimated $48.3 billion annually. Hospitalization accounts for $31.7 billion and fatal brain injuries cost the nation $16.6 billion each year[42]. To put this in perspective, the cost of preventable TBI is second only to nonpreventable AD at $115 billion.

## THE EXTENT OF THE PROBLEM: ETIOLOGY

A bump on the head may have variable consequences depending on previous vulnerabilities (host factors), what kind of injury was sustained, and how the brain reacted to it. Studies of age-specific factors show that the young and the old are high-risk groups[31]. After one brain injury, the risk for a second injury is three times greater; after the second injury, the risk for a third injury is eight times greater[32]. Shaken Baby Syndrome alone affects 1/4000 and carries a death rate of 1/3. Only 1/3 seem to remain without sequelae. Rotational forces are the most deleterious[33]. Athletes in full-contact sports, such as boxing, football, and hockey, are exposed to single and repeated concussions (National Hockey League: recorded 67 concussions in 2001 season; National Football League: football players may be struck in the head 30–50 times per game.) that may result in subdural hematomas, loss of cognitive function, or even death[34]. TBI incidence clusters around specific high-risk behaviors, as the above, and some noncontact-sport activities like snowmobiling[35], heading the ball in soccer[36], or in-line skating[37].

## GENDER AND AGE ARE MODIFIERS

Males, especially young men, are more prone to TBI with immediate and/or delayed sequelae. Males are more likely than females to avoid treatment or minimize the injury, and suffer repeat injury. Recent studies confirm that concussions in young men are under-reported[38]. Young males, post TBI or MTBI are likely to manifest attention deficit/hyperactivity spectrum, while young females after brain injury are more likely to develop labile mood and depression. Older age groups of both genders are more vulnerable to cognitive deficits that present as early dementia. People with residual deficits tend to lack insight, and underestimate and under-report their disabilities[39]. Their friends and physicians do the same, ensuring that their problems go undetected, untreated, or attributed to something else and mistreated.

The causative factors of TBI are further complicated by the fact that direct impact and loss of consciousness need not take place. Sudden rotation is as likely to produce injury. Recent findings demonstrate not only that a gradient of increasing concussion severity is represented by post-traumatic amnesia (PTA) and LOC, but also that measurable neurocognitive abnormalities are evident immediately after injury without PTA or LOC[40]. Recent research suggests that PTA may be a more sensitive predictor of sequelae than LOC[41].

## MECHANISMS OF DAMAGE AND REPAIR

The brain is soft and may sustain injury against sharp boney ridges inside the skull, particularly in the frontal and temporal lobe areas. This process by which the brain case itself damages brain tissue is referred to as drubbing. Contusion against frontal and occipital regions may produce contralateral lesions called contrecoup contusions as the brain accelerates and decelerates against the skull. Damage may also be caused by shearing, as the brain tends to swirl within the brain case. Despite the brain being soft and having a consistency often described as “Jell-O”, not all injuries resulting in a lesion are accompanied by LOC; the location of the lesion is highly relevant. For example, injuries to frontal lobes may be almost asymptomatic, whereas the same trauma to the brain stem may result in immediate LOC and even sudden death. Thus, sequelae of closed head injury, contusion, concussion, and/or edema may be absent, have delayed manifestations, and may even result in death. Diagnosis is further made difficult because there may be no radiologically visible evidence of injury. Therefore, in order to appreciate brain vulnerability, and to understand how neuronal viability is affected following TBI, one needs a rudimentary understanding of the pathophysiology that is responsible for the deleterious effects of head trauma.

The mechanisms of damage include brain swelling, hypoxic damage, diffuse axonal injury resulting from drubbing, and/or cavitation. Diffuse axonal injury and axonal shear are of particular interest because of their distribution over small areas of the brain. They are asymptomatic, but detectable, because accompanying microvascular damage leaves hemosiderin traces that are radio-opaque even after the elapse of time. *Primary brain injury* mechanisms refer to biomechanical forces acting on axons and microvasculature, producing damage by differential accelerations and stretching of structures of different densities (e.g., white matter is more dense than grey matter). These differing densities of brain tissue result in an unequal movement of adjacent areas that results in microtearing and axonal shearing. The result of such tearing is the formation of many microhemorrhages, which disrupt blood supplies in the affected areas of the brain. The result of this type of trauma is axonal swelling, which peaks at 2 h following insult, and eventual axonal disconnection, which ensues 6–12 h after insult. This axonal disconnection eventually leads to Wallerian degeneration and the formation of axonal retraction balls, which appear up to a few days later[42a,42b]. The insidious nature of this type of *primary brain injury* is exemplified by the fact that neuronal degeneration is later accompanied by the depolarization of adjoining neurons and the corresponding release of excitatory amino acids, which together lead to neuroexcitotoxicity. This activation of N-methyl-D-aspartate (NMDA) and other excitatory receptors, accompanied by increased intracellular oxygen radical and calcium ion formation, leads to membrane peroxidation and subsequent DNA fragmentation and, eventually, apoptosis. The death of neurons in these more localized areas leads to the release of cytokines and other mediators of cell injury that activate proinflammatory molecules, such as nuclear factors (NFκB) and interleukins (IL-1β), which are primarily responsible for inducing an inflammatory response. It is through this induction of the inflammatory pathways that the second stage of brain injury is initiated: This second stage of brain injury we may appropriately term *secondary brain injury* and is characterized by inflammation. The defining pathophysiological event associated with *secondary brain injury* is increased permeability of the blood brain barrier. This increased porosity of the blood brain barrier results in microglia of monocyte lineage (10–20% of glial cells) mobilizing to the area, and then forming adhesions followed by phagocytosis. After 2–3 weeks, microglial stars appear and mark the beginning of the fibrillary phase, which is characterized by the enwrapping of dead neurons (neurofibrillary tangles). The extent to which this type of brain damage is manifested depends on the area and types of structures involved. For example, damage may be focal or diffuse, with solitary or multiple areas involved. Intracranial secondary effects include traumatic hematomas, cerebral edema, hydrocephalus, increased intracranial pressure, and infection. Systemic secondary effects include hypoxemia, hypotension, hypercapnia, anemia, hyponatremia, and hypoglycemia. Seizure disorders (focal, generalized, or complex partial) emerge in 2–12% at 5 years. Other sequelae may include motor-sensory deficits and neurobehavioral disturbances. Delayed sequelae are associated with a variety of brain disorders, including PD, AD, and other dementias, which involve chronic inflammatory changes and progression[43]. Recent research calls into question the idea that neuroimaging signals represent viable functional units, however, they do not appear to distinguish between what is effective synaptic inhibition and deactivation that increase and decrease, respectively, cerebral blood flow and glucose consumption. Unfortunately for the patient, clinical implication may be that neuronal degradation continues while imaging shows normal (oxygen/glucose) signals[45].

The prognostic picture is heterogeneous, reflecting complex pathophysiology. It is beyond the scope of this paper to reiterate what is available in standard referenced texts, however, it behooves the treating physician to familiarize himself with diagnosis, treatment, and prognosis[44,47].

## BRAIN RESERVE AND COMPENSATORY MECHANISMS

The observation that organs have reserve capacity is not new. We speak of cardiac reserve, pulmonary reserve capacity, and we know that we can live with one kidney. The concept of brain reserve or neurocognitive reserve, however, is a relatively new one. It was a result of studies on healthy brains, such as the Nun Study[46] and the Centenarian Study[47], where it was noted that the extent of brain damage did not always correlate with cognitive performance[47,48]. It was clear that the brain, of at least some people, could sustain a fair amount of damage before decompensating into symptomatic dysfunction and illness behavior. In some studies, pathology confirmed that preclinical AD was not associated with cognitive impairment or decline, even on measures shown to be sensitive to very mild AD[49]. The lack of effective predictors of the rate of dementia progression extends to the very earliest stages of the disease, including what is often called mild cognitive impairment without dementia. Some have concluded that a new approach to the identification of correlates of rates of progression is needed[50]. We do not know enough about what happens in the brain following minor damages. As in other organs, cellular repair after injury also takes place in the brain, but the brain has difficulty getting rid of by-products and debris resulting from injury-related molecular interactions. Cellular signaling after neuronal injury and the resultant cytokine cascade, microglial response, and cytopathological alterations have the end result of amyloidogenesis. While the beta-amyloid precursor protein (beta-APP) is neuroprotective, its fibril-inducing form, amyloid beta-peptide (A-beta), derivative of beta-APP, is toxic to neuroglia and plays a major role in neurodegenerative changes following a variety of injuries, including trauma and stroke[51]. Apolipoprotein E (ApoE) and its isoforms also mediate neuronal protection, repair, and remodeling. Overexpression of its gene, ApoE4 allele, is not only a major risk factor for AD, but is also associated with poor outcome after TBI[52]. ApoE interacts with A-beta and tau proteins, which in conjunction with deleterious environmental factors, contribute to the etiologic heterogeneity of AD[53]. Damaged axons can serve as a large reservoir of A-beta, which may contribute to A-beta plaque formation after TBI in humans[54]. As with other systems, silent damages will add up to produce organ failure.

## THE EVIDENCE: ANIMAL EXPERIMENTAL STUDIES

Experimental animal studies are repeatable and yield measurable evidence. For example, MTBI that does not produce acute brain tissue injury after a single hit was followed by abnormal accumulation of neurofibrillary material and increased tau protein immunoreactivity in rats[55]. One month after percussion, degenerative changes and accumulation of cytoskeletal proteins were found in hippocampal and cortical areas, and were associated with behavioral changes, such as less-efficient habituation to a new environment[55]. In ApoE-deficient mice, when compared to wild-type controls, MTBI was associated with widespread neuronal degeneration throughout hippocampal subfields and part of dentate gyrus, accompanied by widespread glial fibrillary protein and immunoreactivity throughout the hippocampus[56]. Others have shown that ApoE3 allele is neuroprotective, whereas ApoE4 increases fatalities in transgenic mice with closed head injury[57]. When transgenic mice with overexpression of human A-beta were subjected to controlled concussions, there was marked atrophy of hippocampal and cingulate fields, and a reduction in the deposition of AD-like A-beta. Immunohistochemical studies indicated increased activity and vulnerability to A-beta toxicity[58]. When pigs were subjected to rapid acceleration-deceleration TBI without impact, diffuse axonal pathology with A-beta and tau protein accumulation was observed in damaged fields. Some pigs also exhibited plaque formation typical of AD[59]. The above controlled studies are part of a growing body of evidence showing that MTBI is an epigenetic risk factor for neurodegenerative changes in mammalian AD models.

## HUMAN POSTMORTEM STUDIES

The evidence for intracranial effects of TBI and MTBI in humans is largely circumstantial. However, in humans, as with animal models, increased expression of beta-APP appears to be part of the acute phase response to neuronal injury. Extensive overexpression of beta-APP leads to deposition of beta-amyloid protein and the initiation of an AD-like disease process within days. These findings have implications for the pathogenesis of AD[60]. The first autopsy study to use brain material to study the connection between TBI and AD confirms findings gained from clinical studies[23]. It is well established that deposition of A-beta plays an important role in AD. In fatal TBI, A-beta deposition is associated with the ApoE4 allele[61,62]. TBI induces tau pathology with the formation of neurofibrillary tangles, another major histological marker for AD[63,64,69].

## POPULATION STUDIES

Older epidemiological data support that TBI is a risk factor for subsequent development of AD[24]. In the MIRAGE study, head injury as a risk factor for AD was more strongly associated with subjects completely lacking ApoE4 expression[65]. Saliently, recent longitudinal studies also support significantly increased risk of all dementias and AD in populations with previous TBI[66,67]. The hazard ratio increased as did the severity of TBI. For example, young adults who experienced moderate or severe head injury were found to have more than double the risk of developing AD and other dementias in later life, while the worse the head injury, the higher the risk of AD. For example moderate head injury yielded a 2.3-fold increase in risk; severe head injury more than quadrupled the risk[66]. More recent population-based studies suggest that TBI has long-term implications[68,70a,70b].

## MANAGEMENT ISSUES IN MTBI AND CONCUSSION

Prevention of the second impact syndrome is most important because of rapidly increasing risk, even death[71]. In order to prevent stress-related exacerbations and decompensation, an appreciation of the slow recovery and often-denied residual deficits is a requisite of appropriate management. Detailed protocols for diagnostic grading and treatment have been developed for MTBI/concussion[25,71]. Treatment details are specific to phase of recovery. At first, abstinence from sports and "brain rest" to avoid reinjury and stress factors remain most important. Time taken to recover is usually greatly underestimated with the result that physicians do not allow adequate time out[25]. By 3 months after injury, the 30–50% of the patient group will still have symptoms compared to a control group[72,73] Even well-recovered patients will be susceptible to periodic impairments under conditions of physiological or psychological stress[74,75]. Increased sensitivity to moderate alcohol, sleep deprivation, lengthy travel schedules, and workload can become long lasting, and if present for over 2 years, is often regarded as permanent[76,77]. At 1 year after injury, 10–15% of MTBI patients have not recovered[78,79] and some feel worse. Persistent Post Concussion Syndrome (PPCS) can develop in up to 15% of this high-risk group[72,78,79,80]. Some patients, especially those with demanding jobs (or personalities) and the elderly may always be aware of deficits in performance[76,81].

Antidepressants are indicated in postconcussion depression, the choice dictated by side-effect profile[82]. Hamilton Rating Scale scores, psychomotor speed, verbal memory, recent visual memory, and cognitive efficiency all improve with treatment[83]. ECT remains highly effective and underutilized[84]. More recently, cholinesterase inhibitors used in the treatment of dementia have been found useful in the short-term treatment of cognitive impairment after TBI and, unlike for AD, improvement may be enduring[85,86]. For residual deficits, new rehabilitative techniques have been explored. Brain stimulation, especially novel and unaccustomed physical and mental activity, produces brain-derived nerve growth factors (BDNF) and related neurosteroids that promote cell division, migration, and axonal growth and branching into damaged fields. Novel mental stimuli and other rehabilitative methods akin to mental calisthenics have demonstrated efficacy and have gained popularity as cognitive enhancers and as techniques for brain rehabilitation after injury[87]. Properly focused psychotherapy can help in the functional recovery from PPCS[88]. The "use it or lose it" maxim applies, but forced mental exertion may produce stress response leading to elevated cortisol levels, which counteract the benefits of brain rest.

Unfortunately, we only see the tip of the iceberg. The *silent epidemic* of TBI will continue to be a major contributing factor to a variety of brain disorders manifesting later in the life cycle.
